# Fifteen-Year Evolution of Chronic Cutaneous Leishmaniasis Mimicking Other Dermatoses

**DOI:** 10.4269/ajtmh.25-0575

**Published:** 2026-03-19

**Authors:** Luisa Hurtado-Rossi, Melissa Gutierrez-Gomez, Alvaro J. Martinez-Valencia, Jonny A. García-Luna

**Affiliations:** ^1^ Centro Internacional de Entrenamiento e Investigaciones Médicas (CIDEIM), Cali, Colombia;; ^2^ Department of Internal Medicine, Dermatology Section, Universidad del Valle, Cali, Colombia;; ^3^ Universidad Icesi, Cali, Colombia;; ^4^ Fundación Valle del Lili, Cali, Colombia

## Abstract

Cutaneous leishmaniasis is an endemic parasitic infection in Colombia that typically presents as ulcerated skin lesions, although atypical variants can mimic diverse dermatosis leading to misdiagnosis and delayed treatment. We report the case of a 32-year-old woman with a 15-year history of progressive granulomatous and exophytic tumor-like plaques involving her lower face and neck. She was initially misdiagnosed with lymphocutaneous sporotrichosis and treated unsuccessfully with itraconazole for 1 year. The chronic facial involvement during her formative years resulted in major aesthetic and psychosocial consequences. Histopathology and direct smear ultimately confirmed *Leishmania* spp. infection. She received intramuscular meglumine antimoniate with marked clinical improvement. This case illustrates the diagnostic challenges of atypical chronic cutaneous leishmaniasis and emphasizes the need for thorough evaluation in endemic regions. Early recognition and appropriate management are essential to avoid therapeutic delays and long-term psychosocial impact.

## INTRODUCTION

Leishmaniasis is a vector-borne parasitic infection caused by protozoan parasites of the *Leishmania* species, transmitted in the Americas through the bite of infected female sandflies of the genera *Lutzomyia*. The WHO classifies it as a Neglected Tropical Disease (NTD), with an estimated one billion people living at risk of infection in endemic areas.^1^^,^^2^ Several clinical forms are recognized, of which cutaneous leishmaniasis (CL) is the mildest and most common.^2^ Although patients are generally systemically well, proper diagnosis and treatment are essential because CL can lead to permanent scarring, decreased quality of life, stigmatization, and psychological consequences. Moreover, CL has been described as one of the great mimickers in dermatology, with numerous atypical presentations that can imitate other dermatoses, making the diagnosis challenging and often delayed.^3^

## CASE REPORT

**Figure 1.**
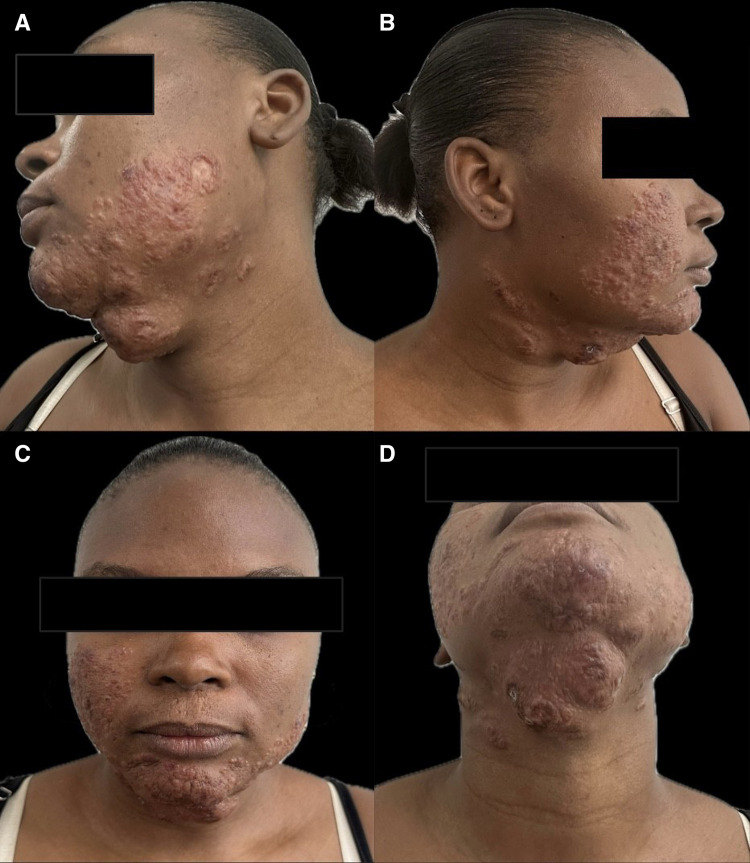
(**A–D**) Clinical presentation at admission showing multiple infiltrated, granulomatous, and exophytic plaques with erythematous borders on the cheeks, chin, and lateral neck, some with ulceration and central crusts.

A case of a of a 32-year-old woman with a 15-year history of progressive cutaneous lesions is presented. The condition was initially presented as a papule on the lower lip, which regressed spontaneously but later reappeared as a similar lesion on the right cheek. Over time, this lesion evolved into an extensive granulomatous plaque with irregular, partially defined borders, involving the right cheek, left mandibular line, and chin. Additional lesions appeared on the lateral neck, along with two exophytic tumor-like plaques with umbilicated centers and central crust located on the mandibular region (Figure 1A–D). There was no mucosal involvement, lymphadenopathy, or hepatosplenomegaly. Systemic evaluation, including complete blood count, renal and hepatic function tests, fasting glucose, and HIV serology, was within normal limits.

The patient was born and lived her entire life in El Charco, Nariño, a rural, highly endemic area for leishmaniasis in southwestern Colombia, until 2015, when she moved to Cali after approximately 5 years of lesion evolution. Her brother had previously developed a localized cutaneous lesion on the leg, treated with traditional plant-based preparations. She had frequent exposure to sylvatic environments, often accompanying relatives to nearby plantations where she recalled experiencing insect bites.

After moving to Cali, the patient sought medical care in a private practice, where a biopsy suggested lymphocutaneous sporotrichosis, with cultures reported as negative. She received multiple empirical treatments, including penicillin-based injections and oral itraconazole for 1 year, without improvement. Over time, she became discouraged due to the persistence of the lesions and the lack of a definitive diagnosis. Approximately 2 years after completing antifungal therapy, she presented to referral centers for further evaluation.

**Figure 2.**
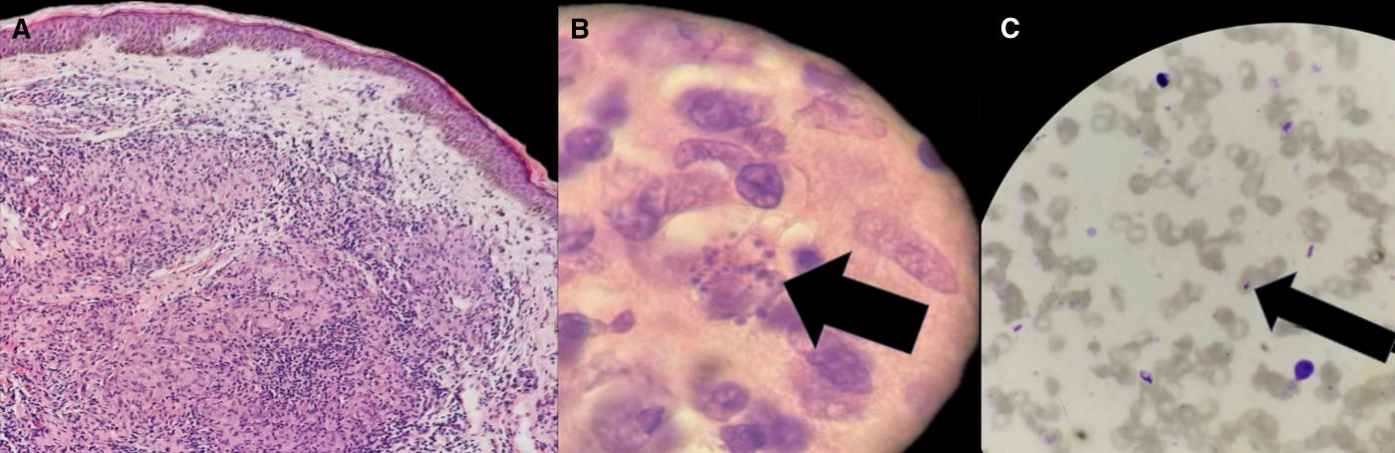
(**A**) Histopathology demonstrating epidermal atrophy and a dense dermal granulomatous infiltrate composed of lymphocytes, plasma cells, histiocytes, and multinucleated giant cells (hematoxylin and eosin stain [H&E], 20×). (**B**) High-power microscopy showing histiocytes with intracellular amastigotes (arrow) (H&E, 100 × oil immersion). (**C**) Direct smear with Giemsa stain highlighting an extracellular amastigote (arrow), with visible nucleus and kinetoplast (40×).

New biopsies were performed for histopathology, special stains, and cultures. Histology demonstrated an atrophic epidermis and granulomatous inflammation composed of lymphocytes, plasma cells, histiocytes, and multinucleated giant cells, within which intracytoplasmic microorganisms were identified (Figure 2A–2B). A direct smear confirmed the diagnosis by demonstrating amastigotes (Figure 2C), establishing a definitive diagnosis of CL.

**Figure 3.**
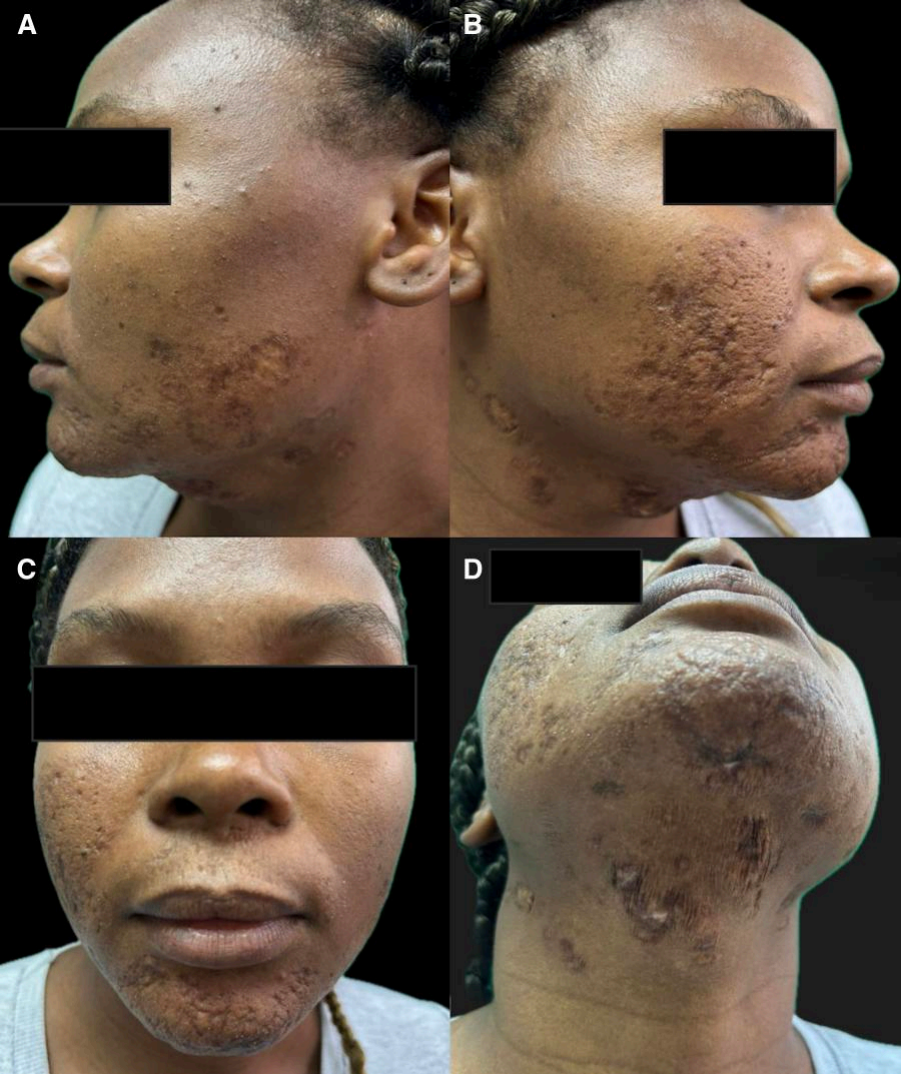
(**A–D**) Follow-up after 13 weeks of treatment, showing marked regression of plaques, decreased infiltration, and residual scar-like hyperpigmented lesions without new activity.

The patient was treated with intramuscular meglumine antimoniate (20 mg/kg/day for 20 days), along with cephalexin (300 mg every 6 hours for 7 days) for secondary bacterial infection. Adherence was complete, with mild adverse events, including myalgias and arthralgias predominantly in the hands and feet and occasional headaches, all of which resolved without sequelae and did not require treatment interruption. At 13-week follow-up, significant improvement was observed, with thinning of the plaques, decreased infiltration, residual atrophic scarring, dyspigmentation, and mild fibrosis over the previously affected areas, without functional limitation or new lesions (Figure 3A–D).

## DISCUSSION

Leishmaniasis is a vector-borne disease caused by protozoan parasites of the *Leishmania* species, transmitted through the bite of infected female sandflies of the genera *Phlebotomus* and *Lutzomyia*. The WHO classifies it as a neglected tropical disease due to its association with long-term disability, stigmatization, and predominance in rural, socioeconomically vulnerable populations with limited access to health care.^1^ The disease is endemic in more than 90 countries, with an estimated 2 million new cases annually and a global prevalence of approximately 12 million.^4^ About 90% of cases occur in a limited number of countries.^4^ In the Americas, the highest burden is reported in Brazil, Colombia, and Peru.^4^^,^^5^

Depending on the *Leishmania* species, the disease manifests mainly as localized cutaneous leishmaniasis (CL), mucocutaneous leishmaniasis (MCL), or visceral leishmaniasis (VL). CL, the most prevalent form worldwide presents with skin ulcers that may be accompanied by satellite lesions or nodular lymphangitis.^4^^,^^6^ In the New World, species such as *L. amazonensis*, *L. chagasi*,* L. mexicana*,* L. naiffi*,* L. braziliensis*, and *L. guyanensis* are common. New World species, particularly those of the *Viannia* subgenus (*L. braziliensis*,* L. guyanensis*), are more often associated with severe, destructive disease and MCL.^7^ In Colombia, 10 *Leishmania* species have been reported, including *L. panamensis* and *L. braziliensis*, which are among the species most often associated with CL.^8^

Although CL may heal spontaneously within 3–18 months, it can leave permanent scars and, in some cases, particularly those caused by *L. braziliensis* and *L. panamensis*, may become chronic or persistent, and approximately 10% progress to more severe forms such as mucocutaneous or disseminated leishmaniasis.^7^^,^^9^ Lesions begin as papules that enlarge into nodules and ulcerate over months, with patients generally systemically well. Forms caused by* L. mexicana* tend to be milder, whereas Viannia species are linked to more extensive ulceration and MCL risk.^7^ CL has been classically divided into localized, diffuse, disseminated, and recidivant forms, but atypical presentations mimic other dermatoses, including erysipeloid, sporotrichoid, eczematous, lupoid, verrucous, paronychial, psoriasiform, and panniculitic forms.^6^^,^^8^ These variants, especially in chronic forms, often delay diagnosis because of their altered clinical appearance and prolonged disease course.^8^

Chronic cutaneous leishmaniasis (CCL) lacks a universally accepted definition, though lesions persisting >2 years are generally classified as chronic.^3^ Exceptionally prolonged cases of CL have been reported, including lesions persisting for 10 years in an immunocompetent host, diffuse disease >30 years in a patient with impaired cell-mediated immunity, and imported *L. infantum* CL manifesting 19 years after exposure. Our patient, with 15 years of progressive localized disease, highlights that chronicity may arise from parasite–host immune interactions as well as social inequities and diagnostic delays, reinforcing the neglected burden of this condition.^10^^–^^12^

Subtypes of CCL include lupoid leishmaniasis, clinically resembling lupus vulgaris, and recidivant leishmaniasis, new lesions arising on or near healed lesions. CCL lesions usually have a low parasite burden, are often resistant to treatment, and may be linked to Th1 immune response dysregulation with increased Th2 cytokines, with chronicity further driven by parasite-induced hyperactivation of the inflammatory response.^7^^,^^13^ Although more frequent in the Old World, CCL also occurs in the New World, particularly in Brazil, Colombia, Peru, and Bolivia, associated with *L. braziliensis*, *L. amazonensis*, *L. panamensis*, and *L. guyanensis*.^14^

This case represents a rare, chronic, sporotrichoid form of CL with 15 years of evolution, initially misdiagnosed as sporotrichosis. Sporotrichoid CL results from the lymphatic spread of amastigotes, producing nodules in a linear arrangement from the primary lesion. This pattern is mostly reported in Brazil and predominantly caused by *L. braziliensis.* Histopathology shows dermal granulomatous inflammation, and the differential diagnosis includes other granulomatous dermatoses.^3^^,^^15^

Granulomatous inflammation is the main histopathological reaction pattern CL, with epithelioid histiocytes and giant cells surrounded by lymphocytes and plasma cells. Granuloma morphology may overlap with other granulomatous dermatoses, explaining the broad differential diagnosis. Sarcoidal granulomas resemble those of sarcoidosis or foreign-body reactions; tuberculoid granulomas with central necrosis mimic cutaneous tuberculosis or leprosy; and suppurative granulomas with neutrophilic micro abscesses are typically seen in deep fungal or atypical mycobacterial infections.^16^

In CL, granulomatous response varies with disease chronicity and parasite burden: diffuse histiocytic infiltrates predominate when amastigotes are abundant, whereas well-formed granulomas with few or no organisms are seen in long-standing lesions. These diverse patterns underscore the diagnostic challenge and the need to correlate morphology with clinical findings and confirmatory tests.^16^

Parasite load is higher in early lesions than in chronic ones but is not associated with lesion size, ulceration, or severity. In CL by *L. braziliensis*, amastigotes are typically scarce in comparison with infections by other species. Amastigotes are mainly found within macrophages but may also occur extracellularly. Interestingly, despite macrophages being the main effector cells for parasite killing, some studies have demonstrated a direct rather than inverse correlation between macrophage density and parasite load. These macrophages exhibit an enhanced inflammatory phenotype but diminished ability to eliminate the parasite, suggesting that persistent antigenic stimulation from residual organisms may amplify the adaptive immune response and perpetuate inflammation.^17^

This mechanism may explain the persistence of lesions in our patient and the long-standing course of the disease. Clinical and histopathological findings were consistent with *L. braziliensis* infection. The predominance of a well-organized granulomatous infiltrate with scarce amastigotes likely contributed to the delay in diagnosis. Chronicity in presumed *L. braziliensis* infection therefore reflects not only the host immune response and low parasite burden but also the diagnostic challenges inherent to late or atypical presentations of CL. Clinicians should consider CL in the differential diagnosis of chronic granulomatous skin diseases, especially in endemic regions.

Species identification was not possible in our patient; however, all species linked to atypical CCL have been reported in Colombia. Diagnosis of CCL is challenging due to low parasite load, frequent negative cultures, and variable polymerase chain reaction (PCR) assay sensitivity.^18^ For this reason, the US CDC recommends combining diagnostic methods such as histopathology, Giemsa staining, culture, and PCR, and obtaining multiple samples to maximize sensitivity.^19^ Histologically, CCL shows atrophic epidermis, dermal lymphocytic infiltrates, granulomas, and scarce amastigotes.^19^^,^^20^ In our patient, repeated tissue sampling with histology and direct smears were required to reach a definitive diagnosis, exemplifying how the multimodal approach is essential in chronic lesions.

CL can cause scarring, stigma, and disability, and it can be complicated by bacterial infection or progression to MCL. Without species identification, treatment should rely on local epidemiology. Systemic therapy is indicated for multiple lesions, facial involvement, or lesions unsuitable for topical therapy. In the New World, intramuscular meglumine antimoniate for 20 days remains first-line for *L. braziliensis* and *L. panamensi*s infections, with early therapy considered the best strategy to prevent progression to CCL.^4^^,^^8^

Beyond the clinical manifestations, CL imposes a substantial social and economic burden, disproportionately affecting women, who are less likely to seek timely medical attention due to limited financial autonomy and prioritization of household needs, factors that contribute to delayed diagnosis and restricted access to care and psychosocial consequences.^21^ Our patient exemplifies this dynamic, having endured years of misdiagnoses before appropriate management, underscoring the intersection of gender, poverty, and health system barriers in endemic regions, and emphasizing the need to address these inequities as part of health equity efforts.

Given the misdiagnosis, our patient received itraconazole for an entire year, in addition to several courses of antibiotics. The cost of itraconazole varies widely across the world, ranging from less than USD $1 per day of treatment to approximately USD $16 per day in Colombia. Moreover, long-term use of itraconazole is not inert and is associated with multiple adverse effects, including hepatotoxicity, pancreatitis, and hormone-related effects, such as gynecomastia, alopecia, decreased libido, hypokalemia, hyponatremia, and even adrenal insufficiency. From a social and economic perspective, this issue is critical for health systems in endemic countries, particularly in settings like Colombia, where limited resources are part of the daily reality of medical practice. Delayed or incorrect diagnoses lead to unnecessary use of expensive and potentially harmful medications, placing additional strain on already overburdened health services.^22^^,^^23^

This case underscores the clinical relevance of recognizing CCL, a rare and underreported entity in the New World, characterized by lesions persisting for 2 years. The patient’s atypical presentation, with unusual extent and morphology, required a thorough clinical and epidemiological assessment to reach the correct diagnosis among multiple differential possibilities. Furthermore, given the extensive facial involvement, a systematic systemic treatment approach was essential to control the disease and prevent further disfigurement.
